# Antioxidant and Anti-Inflammatory Effects of Extracts from Pulsed Electric Field-Treated Artichoke By-Products in Lipopolysaccharide-Stimulated Human THP-1 Macrophages

**DOI:** 10.3390/foods11152250

**Published:** 2022-07-28

**Authors:** Serena Carpentieri, Giuseppina Augimeri, Jessica Ceramella, Adele Vivacqua, Maria Stefania Sinicropi, Gianpiero Pataro, Daniela Bonofiglio, Giovanna Ferrari

**Affiliations:** 1 Department of Industrial Engineering, University of Salerno, Via Giovanni Paolo II, 132, 84084 Fisciano (SA), Italy; scarpentieri@unisa.it (S.C.); gpataro@unisa.it (G.P.); 2 Department of Pharmacy, Health and Nutritional Sciences, University of Calabria, Via Pietro Bucci, 87036 Rende (CS), Italy; giuseppina.augimeri@unical.it (G.A.); jessica.ceramella@unical.it (J.C.); adele.vivacqua@unical.it (A.V.); s.sinicropi@unical.it (M.S.S.); 3 Centro Sanitario, University of Calabria, Via Pietro Bucci, 87036 Rende (CS), Italy; 4 ProdAl Scarl, University of Salerno, Via Giovanni Paolo II, 132, 84084 Fisciano (SA), Italy

**Keywords:** PEF-assisted extraction, artichoke residues, phenolic compounds, bioactivity, HPLC–PDA, human cell lines

## Abstract

In this study, pulsed electric field (PEF—3 kV/cm; 5 kJ/kg) pretreatment was used to intensify the extractability of valuable intracellular compounds from artichoke by-products during a subsequent aqueous extraction (solid–liquid ratio = 1:10 g/mL, T = 20 °C; t = 120 min). Total phenolic content (TPC), antioxidant activity (DPPH, ABTS) and HPLC–PDA analysis of the artichoke extract (AE) and the biological effects on human cell lines were determined. Chlorogenic acid was found to be the most abundant phenolic compound (53% of the TPC) in the AE. The extract showed good antioxidant properties in a concentration-dependent manner. The potential biological effects of AE were investigated using THP-1 macrophages stimulated by lipopolysaccharides (LPS) as an in vitro model system of oxidative stress. Reduced reactive oxygen species production upon treatment with AE was found. Moreover, AE was able to reduce the secretion of the pro-inflammatory mediators Interleukin-6 and Monocyte Chemoattractant Protein-1 in LPS-stimulated macrophages, as determined by qRT-PCR and ELISA assays. These results highlighted the anti-inflammatory and antioxidant properties of the extracts from PEF-treated artichoke by-products, corroborating their potential application as a source of functional ingredients obtained through a feasible and sustainable process.

## 1. Introduction

An integrated global science agenda response which involves food and health is one of the main frameworks for action of the Global Agenda 2030 [1]. Indeed, since unhealthy diets represent the greatest risk to a substantial rise in the incidence of diet-related non-communicable diseases, providing healthy diets from sustainable food systems is an immediate challenge. Currently, among the most widely described and evaluated dietary patterns, the Mediterranean Diet (MD) has been considered one of the healthiest dietary models worldwide, being related to greater longevity and improvement in quality of life [2,3]. The MD recommends the types of foods and the frequencies at which they should be consumed, daily, weekly, and monthly. Vegetables and fruits, whole grains, legumes and nuts are the foods that daily meals should be based on. The beneficial effects of the major components of the MD have been associated with the presence of bioactive compounds. In particular, plant-based foods, including vegetables, are the main source of phenolic compounds and antioxidant nutrients, including vitamins and many minerals, which can protect cells against damage, reducing abnormal processes and even reversing them [4]. In many observational studies, a higher consumption of vegetables has been associated with lower risk of all-cause mortality and morbidity due to cardiovascular disease, Type 2 diabetes, obesity, and cancers [5,6,7,8]. Among vegetables, artichoke is well known due to its nutritional values and therapeutic properties [9]. Interestingly, it has been reported that among the top 50 foods containing the most antioxidants per serving, including red berries, walnuts, cabbage, and broccoli, the artichoke occupies the fourth position of the list, strengthening its competitiveness [10]. Belonging to the Asteraceae family, artichoke (*Cynara scolymus* L. (1753)) is an herbaceous perennial plant that grows in Europe, particularly in Southern Italy, where different varieties are largely cultivated. The edible portion of artichoke is represented by an immature inflorescence (head) composed of tender bracts, and a tasty receptacle, rich in bioactive compounds. The most abundant bioactive molecules are the polyphenols, the concentration of which can be influenced by several factors, such as genotype, physiological stage, agro-technical processes, and the different parts of the plant considered (receptacle, inner and outer bracts, leaves and stems) [11]. In this regard, artichoke processing by-products, including external bracts, and stems, which account for about 60–80% of the total harvested material, are cheap and rich sources of phenolic compounds belonging to the family of hydroxycinnamic acids and flavonoids [12]. Specifically, among the hydroxycinnamic acids, 5-O-caffeoylquinic acid (chlorogenic acid), 1,5-O- and 3,5-O-dicaffeoylquinic acids are the most abundant, while the main flavonoids present in the artichoke residues are apigenin and luteolin glycosides [13,14]. Interestingly, it has been reported that, due to the phenolic content, artichoke extracts not only possess radical scavenging potential, antioxidant, anti-inflammatory, antibiotic and anti-carcinogenic properties [15,16,17,18,19], but also exhibit in vitro inhibitory potential against the main enzymes involved in the onset of metabolic syndromes [20,21,22,23,24].

In recent years, new sustainable green extraction methods have emerged to intensify the extractability of valuable intracellular compounds from plant cell tissues with reduced extraction times and energy and solvent consumption, while preserving the integrity of the major labile extracted bioactives [25,26]. Among them, pulsed electric field (PEF)-assisted solid–liquid extraction of valuable intercellular compounds from plant biomass has been extensively investigated. During PEF treatment, plant biomass in wet form is placed between two electrodes of a treatment chamber and is exposed to an electrical treatment of moderate electric field intensity (0.5–10 kV/cm) and relatively low energy input (1–10 kJ/kg), which induces the permeabilization of cell membranes by pore formation, known as electroporation or electropermeabilization [27]. The PEF-assisted extraction process has shown great potential to selectively recover target intracellular compounds with high yield from a wide range of food-processing wastes and by-products [28], while reducing energy costs, solvent consumption and shortening treatment times [29,30,31,32].

To date, while pressurized liquid extraction and microwave- and ultrasound-assisted extractions have recently been applied to recover dietary fibers and phenolics from artichoke residues [33,34,35], the influence of PEF pre-treatment on the extractability of polyphenols from artichoke residues, namely, involucral bracts, has been investigated only by Battipaglia et al. [36]. However, according to a literature survey, no studies have been carried out so far on the application of PEF-assisted extraction of phenolics from artichoke stems and on the subsequent evaluation of the biological effects of the extracts using human cell lines.

Therefore, the aim of the present study was to explore the potential of extracts obtained through PEF-assisted extraction from artichoke by-products, particularly stems, investigating their biological effects in human lipopolysaccharide-stimulated THP-1 macrophages, with specific emphasis on their antioxidant and anti-inflammatory activities.

## 2. Materials and Methods

### 2.1. Raw Materials and Chemicals

Fresh artichoke stems of the “Cavaliere” variety were provided by a local producer and stored under refrigerated conditions (4 °C) until use. Before each experiment, stems with an initial moisture content (on wet basis) of 90% (*w*/*w*) were cut into pieces of 10 cm length with a knife and then loaded into a pilot dicer machine (Giulio Raiola, Angri, Italy) to obtain 1 cm^3^ cubes. The cubes of artichoke stems were immediately immersed in water with 1% (*w*/*v*) citric acid, prior to further experiments to avoid oxidation. Folin–Ciocalteu reagents, sodium carbonate, citric acid, ABTS, DPPH, Trolox, MTT, LPS, DMSO, methanol, ethanol, sodium phosphate monobasic, sodium phosphate dibasic, sodium chloride, formaldehyde and potassium persulfate were supplied by Sigma-Aldrich (Sigma-Aldrich, Steinheim, Germany). All the standards used for HPLC–PDA analysis were provided by Acros Organics (Geel, Belgium). CellROX^®^ Green Reagent was purchased from Invitrogen (Invitrogen S.r.l., Milan, Italy). An ELISA kit was acquired from Thermo Fisher Scientific (Thermo Fisher Scientific, Waltham, MA, USA). Human THP-1 monocytic cell lines were obtained from the American Type Culture Collection, where they were authenticated, stored according to the supplier’s instructions and used within 4 months after recovery of the frozen aliquots.

### 2.2. PEF-Assisted Extraction Process

PEF treatments of artichoke stems were performed using a laboratory-scale batch system previously described [30]. Briefly, the apparatus consisted of a high-voltage pulsed power (25 kV–500 A) generator (Modulator PG, Scandi Nova, Uppsala, Sweden) capable of delivering monopolar square wave pulses (3–25 µs, 1–450 Hz) to the plant matrix placed between two plane parallel electrodes of stainless steel separated by a Teflon spacer (4 cm electrode gap, 75 cm^2^ electrode area). The maximum electric field intensity (E, kV/cm) and the total specific energy input (W_T_, kJ/kg) were determined, as reported by Bobinaite et al. [37].

The PEF treatments were carried out at the optimal treatment conditions (field strength, E_opt_ = 3 kV/cm; energy input, W_T,opt_ = 5 kJ/kg), identified by analyzing impedance measurement data (data not shown), which allowed us to obtain the maximum level of cell membrane permeabilization with a minimal treatment intensity [38,39]. In each experiment, 60 g of artichoke stem cubes were loaded into the PEF treatment cell, to which distilled water at a constant S–L ratio of 1:1 was added to ensure electrical continuity.

After the electro-permeabilization step, the samples were loaded into 2 L Pyrex flasks, using distilled water as the extracting solvent, at a constant solid–liquid ratio of 1:10 g/mL, as previously described by Battipaglia et al. [36]. The flasks were then placed in an orbital incubator S150 (PBI International, Milan, Italy) to conduct the extraction process at the previously optimized extraction conditions (20 °C for 120 min, under constant shaking at 160 rpm). 

For the sake of comparison, untreated (control) artichoke stem cubes were subjected to a conventional solid–liquid extraction process using the same extraction protocol but without the application of the PEF pre-treatment. 

At the end of the extraction process, the solid was removed and the extract was centrifuged at 5700× *g* for 10 min (PK121R model, ALC International, Cologno Monzese, Milan, Italy) to obtain a clear supernatant. The latter was further concentrated in a R-200/205 Rotavapor (BÜCHI Labortechnik AG, Flawil, Switzerland) set at 35 °C until a decrease in volume of up to 90% was achieved. The concentrated sample was then freeze-dried using a 25 L VirTis Genesis freeze-drier (SP Scientific, Gardiner, NY, USA) at a pressure of 50 mbar for 24 h, by setting the plate temperature at 25 °C. The dry extract was packed under vacuum in plastic–aluminum pouches and stored under refrigerated conditions (8 °C).

### 2.3. Characterization of the Extracts

#### 2.3.1. Proximate Analysis of the Extract

Protein, ash and fat contents were determined according to AOAC Official Methods, specifically AOAC 920.152 (micro-Kjeldahl method, conversion factor 6.25), AOAC 923.03 and AOAC 922.06, respectively. Moisture content was determined through the gravimetric method in an oven (Heraeus Group, Hanau, Germany) at 105 °C until a constant mass was reached (AOAC, 2003). Total dietary fiber was determined using a commercial kit (Megazyme K-TS, Wicklow, Ireland) according to the AOAC 991.43 method. Total carbohydrates were evaluated by subtracting the sum of the percentages of the other proximate components for 100 g of extract [40]. All the analyses were performed in triplicate, and the results were reported on a dry weight basis.

#### 2.3.2. Determination of Total Phenolic Content (TPC) and Antioxidant Activity by DPPH and ABTS

The total phenolic content (TPC) of artichoke stem extracts was determined using the Folin–Ciocalteau method as described by Bobinaite et al. [37], with slight adjustments. Briefly, 1 mL of diluted extract in distilled water (S–L ratio of 1:100 (*w*/*v*)) was mixed with 5 mL of 10% (*v*/*v*) Folin–Ciocalteau reagent and left to rest for 5 min at room temperature. Subsequently, 4 mL of sodium carbonate (7.5%, *w*/*v*) was added to the mixture. The solution was put in the dark for 60 min at room temperature. The absorbance of the solution was then evaluated at 765 nm using a V-650 spectrophotometer (Jasco Inc. Easton, MD, USA). Gallic acid dissolved in water was utilized to create five-point external standard calibration curves (concentration range of 1 to 100 mg/L). The results were expressed as milligrams of gallic acid equivalents per g of freeze-dried artichoke stem extract (mg GAE/gDW). 

The radical scavenging effects of the extract on the 1,1-diphenyl-2-picryl-hydrazil (DPPH) radical were evaluated as described by Fazio et al. [41], with minor corrections. First, the dry extract was dissolved in distilled water to prepare a stock water solution (0.15 g/mL) and from this, through serial dilutions, all the other necessary solutions were obtained (0.001, 0.005, 0.01, 0.02, 0.04, 0.08 g/mL). Then, 20 μL of each prepared extract solution was mixed with 180 μL of a methanol DPPH 0.1 mM in a 48-well plate to obtain seven different concentrations (0.1, 0.5, 1, 2, 4, 8 and 15 mg/mL). The control was prepared by diluting 20 μL of distilled water in 180 μL of DPPH methanol solution. The solution was shaken actively and put in the dark at room temperature for 30 min. The scavenging activity of each solution was measured by means of a spectrophotometer at 517 nm using a microplate reader. DPPH radical scavenging was expressed as percent inhibition (%I) for the extract with respect to the initial concentration of DPPH (control), in agreement with the formula:

%I_DPPH_ = [(Abs_517nm_CTRL − Abs_517nm_ Sample)/(Abs_517nm_ CTRL)] × 100%

The obtained percentages were used to calculate IC_50_ values using GraphPad Prism 9 software (GraphPad Inc., San Diego, CA, USA). Trolox was used as a positive control to calculate the IC_50_ values and as a standard antioxidant compound (0.1–50 µg/mL) to generate a calibration curve (y = −8.9843x + 0.4995, R^2^ = 0.997) for the evaluation of the Trolox equivalent antioxidant capacity (TEAC), expressed as mg Trolox equivalents/g dry matter extract (mg TE/gDM).

The radical scavenging effects of the extract on a 2,2′-azino-bis(3-ethylbenzothiazoline-6-sulfonate) radical cation (ABTS^•+^) were estimated according to the method described by Fazio et al. [41], with some modifications. Briefly, 2 mM ABTS and 70 mM potassium persulfate water solutions were mixed and incubated in the dark for 16 h at room temperature to obtain ABTS^•+^ radical stock solution. Before use, the ABTS solution was diluted in ethanol to obtain an absorbance of 0.70 ± 0.02 at 730 nm. First, the dry extract was dissolved in distilled water to prepare a stock water solution (1.5 g/mL) and, starting from this, through serial dilutions, all the other necessary solutions (0.01, 0.05, 0.1, 0.2, 0.4, 0.8 g/mL). Then, 2 μL of each prepared extract solution was mixed with 198 μL of the ABTS^•+^ solution in a 48-well plate to obtain seven different concentrations (0.1, 0.5, 1, 2, 4, 8 and 15 mg/mL). The solutions obtained were vigorously mixed and incubated at room temperature for 5 min in the dark. The ABTS scavenging activity of each solution was evaluated at 730 nm using a microplate reader. The control was prepared by mixing 2 μL of distilled water with 198 μL of the ABTS^•+^ solution. The ABTS radical scavenging was reported as %I of the extract with respect to the initial concentration of ABTS (control), in agreement with the formula:

%I_ABTS_ = [Abs_730nm_CTRL − Abs_730nm_ Sample)/(Abs_730nm_CTRL)] × 100%

IC_50_ values for the extracts were calculated from the inhibition percentages (%I_ABTS_) using GraphPad Prism 9 software (GraphPad Inc., San Diego, CA, USA). Trolox was used as a positive control to calculate the IC_50_ values and as a standard antioxidant compound (0.1–50 µg/mL) to generate a calibration curve (y = −12.302x + 0.8393, R^2^ = 0.998) for the evaluation of the Trolox equivalent antioxidant capacity (TEAC), expressed as mg Trolox equivalents/g dry matter extract (mg TE/g DM).

#### 2.3.3. HPLC–PDA Analysis of the Extract

The high-performance liquid chromatography–photodiode array detection (HPLC–PDA) analysis for the identification of phenolic compounds in the extracts from both untreated and PEF-treated artichoke stems was performed using a Waters 1525 Separation Module coupled to a photodiode array detector, Waters 2996 (Waters Corporation, Milford, MA, USA), following a modified method reported by Carpentieri et al. [30]. Analytical separation of the identified compounds was carried out in a Waters Spherisorb C18 reverse-phase column (5 μm ODS2, 4.6 × 250 mm, Waters Corporation, Milford, MA, USA).

Before the injection, the artichoke extracts, prepared by dissolving the dry extracts in distilled water (S–L of 1:100 (*w*/*v*)), were filtered with a 0.45 μm filter. The mobile phase was composed of phosphoric acid (0.1%, eluent A) and methanol (100%, eluent B). The separation of the analytes was performed by the following gradient elution: 0–30 min from 5% B to 80% B, 30–33 min 80% B, 33–35 min from 80% B to 5% B. The injection volume and the flow rate of the mobile phase were 5 µL and 0.8 mL/min, respectively. The signal for the quantification of each compound was reported at the wavelength of maximum absorbance λ = 271 nm, 320 nm, 280 nm, 330 nm, 283 nm, 260 nm for gallic acid, chlorogenic acid and cynarine, catechin, epicatechin and phlorizin, sinapic acid, naringin, rutin, respectively. All commercial standards were solubilized into distilled water to create 6-point standard calibration curves (R^2^ = 0.999). The results were expressed as mg of target compounds (gallic acid, chlorogenic acid, catechin, epicatechin, cynarine, sinapic acid, naringin, rutin, phlorizin) per g of freeze-dried artichoke stem extract (DW). 

### 2.4. Cell Viability Assay

For the cell viability assay, 2.2 × 10^4^ THP-1 were seeded into 24-well plates and differentiated into M0 macrophages, as described by Gionfriddo et al. [42]. M0 macrophages were exposed for 1 h to 1 and 2 mg/mL of artichoke extract, dissolved in distilled water, prior to treatment for 24 h with 10 ng/mL of lipopolysaccharides (LPS) capable of polarizing M0 (negative control) into the inflammatory M1 macrophages (positive control). The 3-(4,5-dimethylthiazol- 2-yl)-2,5-diphenyltetrazolium (MTT) assay was used to evaluate the cell viability, as previously described [43]. The absorbance was measured at a test wavelength of 570 nm in a Multiskan SkyHigh Photometer (Thermo Fisher Scientific, Milan, Italy). The data were reported as the percentage of viable cells compared to the control (100%). 

### 2.5. Real-Time RT-PCR Assays

From cells treated according to the experimental conditions reported in Section 2.4, total RNA was extracted as previously reported by Giordano et al. [44]. Using SYBR Green Universal PCR Master Mix, 2 μL of diluted (1:10) cDNA was determined in duplicate by real-time PCR, analyzed in an iCycler iQ Detection System (Bio-Rad, Hercules, CA, USA), with 18S mRNA content being used to normalize each sample. Relative gene expression levels were calculated as previously described by Augimeri et al. [45]. The primers used for the gene amplification are listed in Supplementary Table S1.

### 2.6. Enzyme-Linked Immunosorbent Assay (ELISA)

Interleukin (IL)-6 and Monocyte Chemoattractant Protein (MCP)-1 levels were measured in supernatants from M0 macrophages (negative control) exposed for 1 h to 1 and 2 mg/mL of artichoke extract, dissolved in distilled water and then treated with 10 ng/mL of lipopolysaccharides (LPS) for 24 h. M0 macrophages treated with LPS were used as positive controls. IL-6 and MCP-1 concentrations were detected using the ELISA kit (Thermo Fisher Scientific, Waltham, MA, USA). The results were presented as pg/mL. 

### 2.7. Measurement of Intracellular Reactive Oxygen Species Production

M0 macrophages (negative control) were exposed to artichoke extracts at concentrations of 1 and 2 mg/mL prior to treatment with 1 μg/mL of lipopolysaccharides (LPS) for 3 h. M0 macrophages treated with LPS were used as positive controls. Intracellular reactive oxygen species (ROS) were detected by staining with CellROX^®^ Green Reagent 5 μM for 30 min at 37 °C, which allowed the detection of oxidative stress. After washing with PBS, cells were fixed with 3.7% formaldehyde for 15 min. The levels of intracellular ROS were evaluated under an Olympus BX51 fluorescence microscope (Olympus, Tokyo, Japan) equipped with a 10× objective lens and quantified using ImageJ. The data were reported as the mean pixel intensity normalized to number of cells. 

### 2.8. Statistical Analysis

All the experiments were carried out in triplicate and the results were shown as means ± standard deviations (SDs) or standard errors of the mean (SEMs). Differences between mean values were analyzed with parametric tests (one-way ANOVA with Tukey’s post hoc test for the analysis of the concentrations of phenolic compounds by HPLC–PDA, one-way ANOVA with Dunnett’s post hoc test for the analysis of the antioxidant effects and Student’s *t*-test for the analysis of the antioxidant and anti-inflammatory effects in cell cultures). Statistical significance was set at *p* < 0.05. 

## 3. Results

### 3.1. Proximate Composition of the Extracts

Table 1 shows the proximate analysis on dry basis of the lyophilized artichoke extract (AE) obtained upon water extraction of PEF-treated artichoke stems. A total dietary fiber content of 19.62 ± 1.21 g/100 g dry weight (gDW) and a carbohydrate content of 66.67 ± 1.24 g/100 gDW were measured. It is worth noting that the protein content (9.48 ± 0.92 g/100 gDW) was similar to that previously reported for different artichoke by-products and their extracts (10.35–14.7 g/100 gDW) [35,46,47,48]. In contrast, the fat content (0.42 ± 0.06 g/100 gDW) was lower than that typically found by other authors for artichoke by-products (1.30–3.7 g/100 gDW) [46,48], representing an additional positive aspect for the potential use of this extract as an ingredient in the formulation of functional products [49].

### 3.2. Total Phenolic Content, Antioxidant Activity and HPLC–PDA Analysis of the Extracts

**Figure 1:**
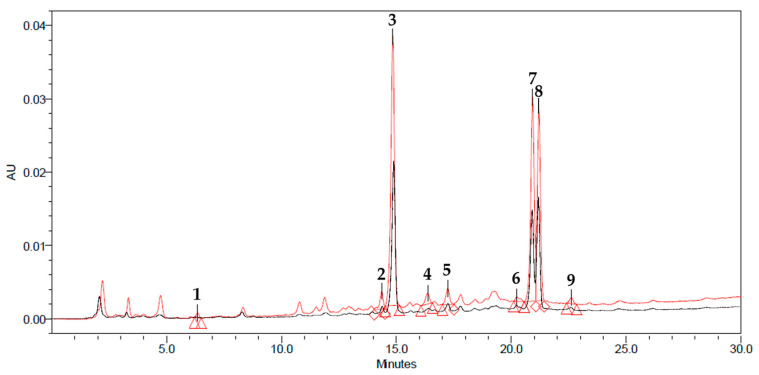
HPLC–PDA chromatograms of the extract from untreated artichoke stems (black line), and the extract from PEF-treated artichoke stems (red line). Peak identification: gallic acid (**1**), catechin (**2**), chlorogenic acid (**3**), epicatechin (**4**), cynarine (**5**), sinapic acid (**6**), naringin (**7**), rutin (**8**), phlorizin (**9**).

The total phenolic content (TPC) and the antioxidant activity of the AEs from untreated and PEF-treated artichoke stems, evaluated via Folin–Ciocalteu, DPPH and ABTS assays were 7.03 ± 1.08 mg GAE/gDW, 4.65 ± 1.4 mg TE/gDW and 6.09 ± 3.0 mg TE/gDW for the untreated sample, and 13.63 ± 1.08 mg GAE/gDW, 9.1 ± 1.4 mg TE/gDW and 13.3 ± 3.0 mg TE/gDW for the PEF-treated one. It is worth noting that the TPC values detected for the extracts were in agreement with those found by other scientists, with slight differences, depending on the artichoke variety, the type of residue and the experimental protocol. For instance, the TPCs of extracts from artichoke sprouts and stalks from different Italian locations detected by Ferioli and D’Antuono were in the range of 8.9–34.9 mg GAE/gDW [50]. Similarly, Rejeb et al. found the TPCs of extracts from artichoke floral stems to be equal to 10.689 ± 0.324 mg GAE/gDW [23]. Moreover, the composition, in terms of the most abundant phenolic compounds, of the extracts from untreated and PEF-treated artichoke stems subject to optimal processing conditions was assessed via HPLC–PDA analysis. The resulting chromatograms, depicted in Figure 1, showed that the two extracts presented similar chromatogram profiles and that PEF pre-treatment of artichoke stems remarkably increased the peak areas of the major phenolic compounds present in the AEs, corresponding to chlorogenic acid (peak 3), followed by naringin (peak 7), rutin (peak 8) catechin (peak 2), epicatechin (peak 4), sinapic acid (peak 6), phlorizin (peak 9), cynarine (peak 5) and gallic acid (peak 1). The obtained results are consistent with previous findings by other authors [18,23]. They reported that, among phenolic compounds, caffeoylquinic acids were the most abundant hydroxycinnamic acids in artichoke residues, with chlorogenic acid being present to a greater extent. Specifically, as corroborated by the results presented in Table 2, the PEF-assisted extraction process led to a final content of chlorogenic acid in the extract of 6.02 ± 0.63 mg/gDW (+28% compared to the control sample), representing about 93% of the phenolic acids identified in the extract. The values of chlorogenic acid concentration observed in this work were in agreement with the values reported by other authors, with slight differences, depending on the variety, geographical location, the different parts of the artichoke examined, different growing stages, ripening and extraction protocols [51]. As an example, Francavilla et al. [33] and Mena-Garcia et al. [34] found that chlorogenic acid (5-O-caffeoylquinic acid) was the most abundant caffeoyl derivative identified in artichoke stalks extracts (2.79 mg/gDW and 2.39 mg/gDW, respectively). Additionally, in agreement with previous studies [38,46,52,53,54], it could be speculated that phenolic compounds likely contributed to the detected antioxidant activity of the extracts (9.1 ± 1.4 mg TE/gDW and 13.3 ± 3.0 mg TE/gDW).

**Figure 2:**
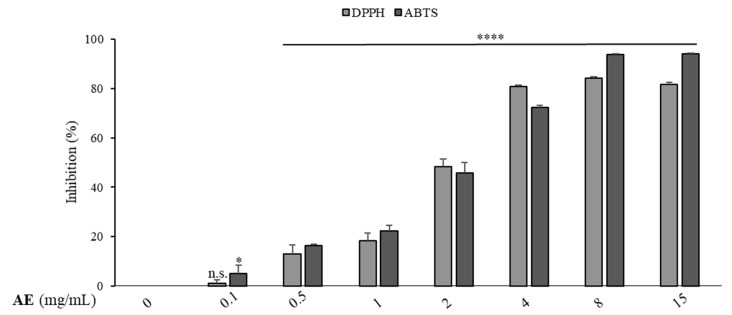
Changes in %I_DPPH_ and %I_ABTS_ of AE at different concentrations. Extract I% vs. initial concentration of ABTS or DPPH (0). The values represent the means ± SDs of three different experiments, each performed in triplicate. n.s.: not significant; * *p* < 0.05; **** *p* < 0.0001.

To evaluate the antioxidant power of artichoke extract (AE), ABTS and DPPH assays were also performed for different AE concentrations (ranging from 0.1 to 15 mg/mL). The results obtained allowed us to conclude that the antioxidant properties of the extract are concentration-dependent, as shown in Figure 2. 

The extract inhibited the radical DPPH by 80.74% at the concentration of 4 mg/mL, reaching the 84% inhibition (%I) at the highest concentrations used in this assay (8 and 15 mg/mL). The capacity for inhibiting the ABTS^•+^ radical cation was 72% after the exposure to 4 mg/mL of the extract and about 94% after exposure to 8 and 15 mg/mL of the extract. The IC_50_ values were calculated from the measured inhibitions against both DPPH and ABTS radicals and are reported in Table 3. The IC_50_ value of the extract against the DPPH radical was 2.4 ± 0.5 mg/mL. The data highlighted that AE exhibited a similar scavenging activity toward the ABTS^•+^ radical cation, with an IC_50_ value of 2.0 ± 0.2 mg/mL. Thus, the extract had a comparable ability to inhibit the radicals DPPH and ABTS by 50%. Finally, the trolox equivalent antioxidant capacity (TEAC) was also calculated for both the methods from the AE concentration of 2 mg/mL, obtaining values of 9.1 ± 1.4 and 13.3 ± 3.0 mg TE/g DM for DPPH and ABTS assays, respectively.

### 3.3. Antioxidant Power of Artichoke Extract in LPS-Stimulated Human Macrophages

**Figure 3:**
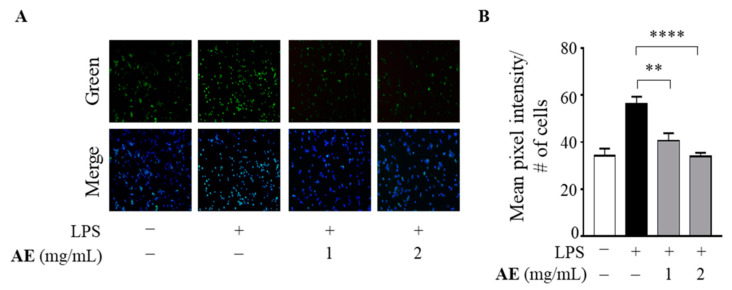
Reactive oxygen species production in lipopolysaccharide-stimulated human macrophages upon treatment with artichoke extract. (**A**) Representative fluorescent microscopy images of reactive oxygen species (ROS) detection in THP-1 macrophages untreated (−) or treated with 1 and 2 mg/mL of artichoke extract (AE) for 1 h and then stimulated with 1 μg/mL of lipopolysaccharides (LPS) for 3 h. Merged fluorescent images of green (CellROX dye) and DAPI (4′,6-diamidino-2-phenylindole) staining of DNA (blue) are shown. (**B**) Quantitative analysis of (**A**) shown as mean pixel intensity normalized to number of cells (#). Data bars are means ± SEMs of two independent experiments. ** *p* < 0.005, **** *p* < 0.0001.

Based on the IC_50_ values of the AE against DPPH and ABTS radicals, we assessed the antioxidant effects of the extract at 1 and 2 mg/mL on human THP-1 macrophages stimulated by LPS into M1 macrophages as an in vitro model system of oxidative stress able to produce reactive oxygen species (ROS) [55]. As shown in Figure 3, LPS increased the production of intracellular ROS in human M1 macrophages, whereas AE at both concentrations significantly inhibited the ROS production induced by LPS (*p* < 0.005, *p* < 0.0001, respectively).

**Figure 4:**
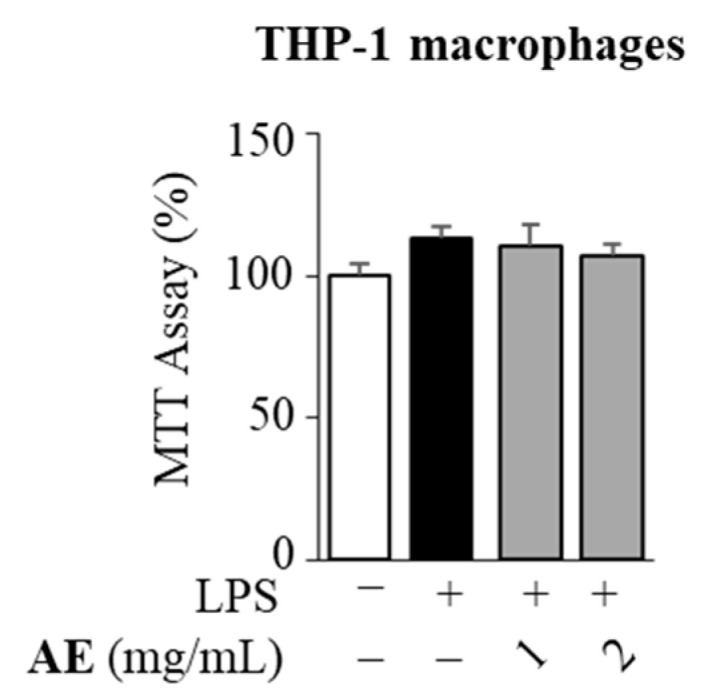
MTT (3-(4,5-Dimethylthiazol-2-yl)-2,5-Diphenyltetrazolium Bromide) growth in lipopolysaccharide (LPS)-stimulated THP-1 macrophages untreated (−) or treated for 24 h with 1 and 2 mg/mL of artichoke extract (AE). Cell viability is expressed as % of control (−). The value represents the means ± SEMs of three different experiments, each performed with triplicate samples.

In order to exclude any cytotoxic effects on cells upon treatment with AE, its biological effects were assessed in LPS-stimulated macrophages by MTT assay. The exposure of cells for 24 h to AE, at concentrations of 1 and 2 mg/mL, did not hamper cell viability (Figure 4). 

### 3.4. Anti-Inflammatory Effects of Artichoke Extract in LPS-Stimulated Human Macrophages

**Figure 5:**
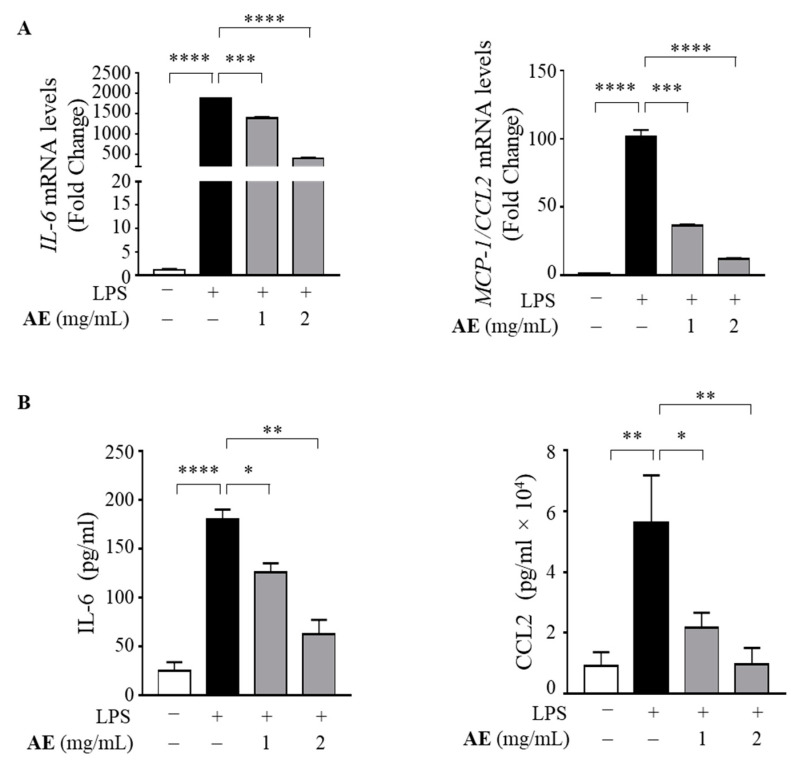
Anti-inflammatory effects of artichoke extract in M1 macrophages. (**A**) Real Time RT-PCR assay for interleukin-6 (IL-6), Monocyte Chemoattractant Protein (MCP)-1/C-C motif chemokine ligand 2 (CCL-2) mRNA expression in human THP-1 derived macrophages untreated (−) or treated for 1 h with artichoke extract (AE) at concentrations of 1 and 2 mg/mL and then stimulated with 10 ng/mL of lipopolysaccharides (LPS) for 24 h. (**B**) Enzyme-linked immunosorbent assay (ELISA) for IL-6 and CCL-2 in human THP-1 derived macrophages untreated (−) or treated as in (**A**). The values represent the means ± SEMs of three different experiments, performed in duplicate. * *p* < 0.05, ** *p* < 0.005, *** *p* < 0.001, **** *p* < 0.0001.

To investigate the potential anti-inflammatory effects of AE, the gene and protein expression levels of pro-inflammatory cytokines were measured by qRT-PCR and ELISA, respectively, in human M1 macrophages treated with 1 and 2 mg/mL of AE. As expected, the treatment with LPS induced the expression of Interleukin (IL)-6 and Monocyte Chemoattractant Protein (MCP)-1/C-C motif chemokine ligand 2 (CCL-2) in M1 macrophages, whereas AE was able to dampen the inflammation in a concentration-dependent manner (Figure 5A). Consistently with these results, it was also found that AE reduced the IL-6 and MCP-1 protein secretion induced by LPS, suggesting its anti-inflammatory effect in macrophages (Figure 5B).

## 4. Discussion

The results of the present study highlighted that the extract obtained from PEF-treated artichoke stems had a high content of phenolic compounds and displayed anti-inflammatory and antioxidant activities in human macrophages. Firstly, from the determination of the chemical composition of the artichoke extract (AE), it was found that AE was characterized by low dietary fiber and high carbohydrate contents (19.62 g/100 gDW and 66.84 g/100 gDW, respectively), in line with the findings of Rabelo et al. [35] for extracts from artichoke bracts obtained using ethanol as an extracting solvent (17.56 g/100 gDW and 47.34 g/100 gDW, respectively). A relatively high protein content (9.48 g/100 gDW) and a low-fat content (0.42 g/100 gDW) were also detected in AE. Thus, besides its high nutritional value, AE can be seen as a valuable fat-free ingredient with potential health-beneficial properties, which could contribute to the prevention of diet-related non-communicable diseases [46,48]. Indeed, ingredients with no more than 0.5 g of fat per 100 g are considered fat-free ingredients according to the Codex Guidelines for nutrition labelling [56]. Additionally, the potential use of this extract as a functional ingredient was confirmed and strengthened, considering the high level of phenolic compounds and antioxidant activity detected in this study. Despite the wide range of factors that affect the type and amounts of phenolic compounds in artichoke by-product extracts (variety, part of the plant, cultivation conditions, harvesting, extraction method and conditions), our findings are consistent with those of other authors reported in previous studies. The values of total phenolic content (TPC, 8.13–35.83 mg GAE/gDW) reported by Zazzali et al., Quispe et al. and Zuorro et al. [57,58,59] for extracts obtained using water or ethanol from by-products of different artichoke cultivars were similar to those (TPC, 13.63 mg GAE/gDW) detected in this work in relation to PEF-assisted extraction. Similarly, Mena-García et al. [34] found that microwave-assisted extraction with an ethanol–water mixture (50:50, *v*/*v*) from artichoke bracts led to extracts with TPCs ranging from 9 to 18 mg GAE/gDW. However, Kollia et al. [60] reported lower values for the TPCs of extracts from artichoke stems (0.33 ± 0.01 mg GAE/gFW) obtained using ultrasound-assisted extraction and a methanol–water mixture (80:20, (*v*/*v*)). The detected values for the TPC and antioxidant activity of the extracts from untreated and PEF-treated artichoke stems allow us to conclude that the application of this extraction procedure, involving the combined use of PEF technology and water as a green extracting solvent, can be considered an effective and sustainable method for valorizing artichoke residues (+103% on average in TPC and antioxidant activity with respect to the AEs from untreated samples). This is in line with the findings of Battipaglia et al. [36] demonstrating that a PEF pre-treatment (1.6 kV/cm, 5 kJ/kg) of artichoke bracts was particularly suitable for the recovery of polyphenols, using water as an extracting solvent, with a significant increase (+150%) in polyphenol yield with respect to the untreated samples. Moreover, Zazzali et al. [59] and Maietta et al. [61] successfully demonstrated that water is a suitable solvent for the extraction of phenolic compounds from artichoke by-products when compared with water–ethanol mixtures. The effective extraction of phenolic compounds from PEF-treated artichoke stems compared to the untreated ones was also confirmed by HPLC–PDA chromatograms, revealing that no degradation phenomena occurred upon the application of PEF. A variety of phenolic compounds, mainly phenolic acids and flavonoids, which are the two major phenolic classes present in artichokes, were identified [11]. Indeed, 48.5% of the total phenolic compounds identified by HPLC–PDA analysis was represented by phenolic acids and derivatives (chlorogenic acid, sinapic acid, gallic acid, cynarine), while the remaining 51.5% were flavonoids (naringin, rutin, catechin, epicatechin, phlorizin). The phenolic profile of the extract revealed high amounts of caffeoylquinic acids, mainly chlorogenic acid, which is in agreement with previous studies addressing the identification of the phenolic compositions of extracts from artichoke bracts, stems and receptacles [9,18,34,46,48,62,63]. The concentration of chlorogenic acid measured in this study represented about half of the total phenolic composition detected in the extract (45%), which is slightly higher than that reported by Noriega-Rodriguez et al. [46], Lattanzio et al. [9] and Schutz et al. (35% on average) [63]. It was widely demonstrated that caffeoylquinic acids, especially chlorogenic acid, are able to exert beneficial effects on human health, including antioxidant properties [64,65,66,67,68,69].

The values for antioxidant activity found in this study (9.1 ± 1.4 mg TE/gDW and 13.3 ± 3.0 mg TE/gDW, for DPPH and ABTS, respectively) are fairly consistent with those reported by other authors (26.59–38.68 mg TE/gDW) [34]. In contrast, as already mentioned in the case of TPC, lower values of DPPH (0.11 ± 0.04 mg TE/gFW) of extracts from artichoke bracts were reported by Kollia et al. [60], which discrepancy may be due to the higher content of chlorogenic acid present in the extract obtained in this study. Indeed, a strong correlation between antioxidant activity and polyphenol content has been extensively demonstrated, as above reported. Thus, these data confirm the potential application of the extract from PEF-treated artichoke stems as a natural promising functional ingredient as well as a nutraceutical or pharmaceutical compound. The regular consumption of antioxidant-rich foods, such as fruits and vegetables, is encouraged by healthy dietary patterns, including the Mediterranean Diet, in order to ensure an optimal antioxidative status, which prevents the development of diet-related non-communicable diseases [70]. Oxidative stress is caused by an imbalance in the production of ROS and the biological antioxidant defense system. The increased production of ROS can promote the expression of key proinflammatory cytokines, such as Tumor Necrosis Factor-α, IL-1β, IL-6 and CCL2/5, which sustain inflammation [71]. It is well known that low-grade chronic inflammation is a risk factor for the development of several chronic pathological conditions, including cardiovascular disease and cancer, which ultimately can lead to patient death. Therefore, reducing systemic inflammatory processes through natural anti-inflammatory agents which impair cytokine production may prevent or delay the onset of chronic illness. Using an in vitro model of cellular oxidative stress and inflammation represented by LPS-stimulated human THP-1 macrophages, it was found that AE reduced the ROS production as well as the gene expression and protein secretion of IL-6 and CCL2, suggesting both the antioxidant and anti-inflammatory properties of AE.

## 5. Conclusions

Our data demonstrated that PEF-assisted aqueous extraction of artichoke by-products allowed the obtainment of extracts rich in phenolic compounds with health-beneficial properties. It can be highlighted that this combined process, together with the utilization of green solvents, can be considered a sustainable procedure for the valorization of vegetable-based biomass, providing high value-added compounds to be exploited for applications in the food, cosmetic and pharmaceutical fields. In particular, the obtained natural extracts, rich in antioxidants, could be suitable to be incorporated into food products for their functionalization. Nowadays, the food industry is striving to meet the needs of consumers who are more and more worried about their health and wellbeing and demanding green labels and natural ingredients. Therefore, the exploitation of artichoke by-products as natural and low-cost sources and the incorporation of their health-promoting extracts into staple foods, such as bread or bakery products, could be a good opportunity to make these ingredients acceptable to consumers and to encourage healthier lifestyles.

## Figures and Tables

**Table 1:** Chemical composition of the extract obtained from PEF-treated artichoke stems.

Moisture content	0.17 ± 0.02
Ash content	3.64 ± 0.21
Protein content	9.48 ± 0.92
Fat content	0.42 ± 0.06
Carbohydrates	66.67 ± 1.24
Total dietary fiber	19.62 ± 1.21

Results are expressed as g/100 gDW on a dry basis. Data are expressed as means (*n* = 3) ± SDs.

**Table 2:** Concentrations (mg/gDW) of gallic acid, catechin, chlorogenic acid, epicatechin, cynarine, sinapic acid, naringin, rutin and phlorizin (HPLC/PDA analysis) in the extracts from untreated and PEF-treated artichoke stems.

Peak No.	Compound	Max Absorption Wavelength (nm)	Retention Time (min)	Concentration (mg/gDW)	
				Untreated	PEF-Treated
1	Gallic acid	271	6.35	nd	0.02 ± 0.003
2	Catechin	280	14.36	0.35 ± 0.03 _a_	0.89 ± 0.06 _b_
3	Chlorogenic acid	320	14.83	4.70 ± 0.37 _a_	6.02 ± 0.63 _b_
4	Epicatechin	280	16.36	0.10 ± 0.01 _a_	0.39 ± 0.07 _b_
5	Cynarine	320	17.24	0.09 ± 0.01 _a_	0.15 ± 0.03 _b_
6	Sinapic acid	330	20.24	0.16 ± 0.02 _a_	0.31 ± 0.04 _b_
7	Naringin	283	20.93	2.06 ± 0.08 _a_	3.46 ± 0.36 _b_
8	Rutin	260	21.32	1.25 ± 0.06 _a_	1.96 ± 0.07 _b_
9	Phlorizin	280	22.61	0.04 ± 0.002 _a_	0.20 ± 0.02 _b_

Data are expressed as means (*n* = 3) ± SDs. Values with different lowercase letters within the same row are significantly different (*p* ≤ 0.05). nd: not detected.

**Table 3:** IC_50_ values of the extract and Trolox, used as positive control, against DPPH and ABTS radicals.

	IC_50_	
Sample	DPPH	ABTS
Artichoke extract	2.4 ± 0.5 mg/mL	2.0 ± 0.2 mg/mL
Positive control		
Trolox	21.6 ± 2.9 µg/mL	26.4 ± 2.5 µg/mL

Data are expressed as means ± SDs (*n* = 3).

## Data Availability

Data is contained within the article or supplementary material.

## Supplementary Materials

The following supporting information can be downloaded at: https://www.mdpi.com/article/10.3390/foods11152250/s1, Table S1: Oligonucleotides used in this study.
