# Supplementary material for: Antioxidant and Anti-Inflammatory Effects of Extracts from Pulsed Electric Field-Treated Artichoke By-Products in Lipopolysaccharide-Stimulated Human THP-1 Macrophages
Source: Foods. 2022 Jul 28;11(15):2250. doi: 10.3390/foods11152250 (PMC9368542; doi:10.3390/foods11152250)
Supplement: Supplementary file 1 [file foods-11-02250-s001.zip › foods-1784627-supplementary.pdf]

Supplementary Table S1. Oligonucleotides used in this study.

Gene Name	Gene Symbol	Species	Primer Sequences	
18s rRNA	*18S*	Human	Forward	5′-CCCACTCCTCCACCTTTGAC-3′
			Reverse	5′-TGTTGCTGTAGCCAAATTCGTT-3′
Interleukin 6	*IL-6*	Human	Forward	5′-CCAGGAGCCCAGCTATGAAC-3′
			Reverse	5′-CCCAGGGAGAAGGCAACTG-3′
Monocyte Chemoattractant Protein-1	*MCP-1* /*CCL-2*	Human	Forward	5′-CAGCCAGATGCAATCAATGCC-3′
			Reverse	5′-TGGAATCCTGAACCCACTTCT-3′
